# Sex differences in mechanical allodynia: how can it be preclinically quantified and analyzed?

**DOI:** 10.3389/fnbeh.2014.00040

**Published:** 2014-02-13

**Authors:** Lauren Nicotra, Jonathan Tuke, Peter M. Grace, Paul E. Rolan, Mark R. Hutchinson

**Affiliations:** ^1^Department of Pharmacology, Neuroimmunopharmacology, The University of AdelaideAdelaide, SA, Australia; ^2^Faculty of Engineering, School of Mathematical Sciences, Computer Science and Mathematics, The University of AdelaideAdelaide, SA, Australia; ^3^Department of Psychology and Neuroscience, The University of ColoradoBoulder, CO, USA; ^4^Department of Physiology, Neuroimmunopharmacology, The University of AdelaideAdelaide, SA, Australia

**Keywords:** neuropathic pain, von Frey, sex, oestrus cycle

## Abstract

Translating promising preclinical drug discoveries to successful clinical trials remains a significant hurdle in pain research. Although animal models have significantly contributed to understanding chronic pain pathophysiology, the majority of research has focused on male rodents using testing procedures that produce sex difference data that do not align well with comparable clinical experiences. Additionally, the use of animal pain models presents ongoing ethical challenges demanding continuing refinement of preclinical methods. To this end, this study sought to test a quantitative allodynia assessment technique and associated statistical analysis in a modified graded nerve injury pain model with the aim to further examine sex differences in allodynia. Graded allodynia was established in male and female Sprague Dawley rats by altering the number of sutures placed around the sciatic nerve and quantified by the von Frey test. Linear mixed effects modeling regressed response on each fixed effect (sex, oestrus cycle, pain treatment). On comparison with other common von Frey assessment techniques, utilizing lower threshold filaments than those ordinarily tested, at 1 s intervals, appropriately and successfully investigated female mechanical allodynia, revealing significant sex and oestrus cycle difference across the graded allodynia that other common behavioral methods were unable to detect. Utilizing this different von Frey approach and graded allodynia model, a single suture inflicting less allodynia was sufficient to demonstrate exaggerated female mechanical allodynia throughout the phases of dioestrus and pro-oestrus. Refining the von Frey testing method, statistical analysis technique and the use of a graded model of chronic pain, allowed for examination of the influences on female mechanical nociception that other von Frey methods cannot provide.

## Introduction

It is now well recognized that sex differences exist in chronic pain pathologies (Ruau et al., 2012). Clinical and experimental studies have demonstrated that women are overrepresented in numerous chronic pain conditions compared to males (Riley et al., 1998; Hurley and Adams, 2008; Fillingim et al., 2009). Animal studies have replicated the clinical pain experience, with female rodents characteristically exhibiting lower thresholds to painful stimuli and exaggerated pain responses following nerve injury compared to males (Lacroix-Fralish et al., 2006a). Evidently, chronic pain is predominantly a female problem.

Neuropathic pain is one chronic pain condition with a predominant number of female sufferers (Riley et al., 1998; Fillingim et al., 2009). Neuropathic pain is specifically is defined as damage or inflammation of the peripheral nervous system, characterized by both hyperalgesia, exaggerated pain in response to noxious stimuli; and allodynia, pain in response to normally innocuous stimuli (Woolf, 1995). Recent preclinical investigations have provided for a greater understanding of the initiation and maintenance processes underlying neuropathic pain. However, due to the continued use of suboptimal preclinical methodologies, substantial mechanistic questions remain, with the reasons for the female prevalence of this chronic pain condition not yet fully understood. Contributing to the translational issues associated with preclinical pain studies, many investigations examining chronic pain mechanisms have done so utilizing animal models, such as chronic constriction injury (CCI), partial sciatic nerve ligation (PSNL) and L5 spinal nerve ligation that do not in fact best reflect the clinical heterogeneity of clinical pain (Wang and Wang, 2003; Mogil, 2009; Grace et al., 2010; Berge, 2011). Consequently, in order to draw improved information from preclinical studies and extrapolate those findings to the clinical situation it is imperative for future studies to develop and utilize novel animal pain models and behavioral testing techniques that more closely resemble clinical chronic pain and to standardize such procedures to enable suitable comparisons and improved interpretation of results thereby allowing appropriate future investigation into the role of sex in chronic pain pathologies (Bove, 2006).

In addition to the various translational issues associated with utilizing animal chronic pain models, the use of animals in pain research poses numerous ethical dilemmas. Inflicting pain in order to ultimately understand and prevent it is the ethical paradox the medical and scientific community have largely accepted in order to validate intentional animal suffering (Zimmermann, 1986; Dubner, 1987; Tannenbaum, 1999). In the interest of protecting the welfare of animals used in scientific research the deliberate infliction of pain in animals requires strong justification (Carbone, 2012). This stringency is transparent throughout the various global statutory policies that govern the use of animals in science. Embedded within this legislation are the guiding principles of the three R's, which form a structure ensuring animals are only used when absolutely necessary (Replacement), as few animals are used as required to achieve the scientific and statistical objectives of the investigation (Reduction) and the models and testing procedures reduce or preclude potential harm, pain and distress (Refinement) (Gad, 1990). Despite easily satisfying the replacement parameter of the three R's with strong evidence for a reliance on and a necessity to use animals in pain research (Mogil et al., 2010), scientists are faced with great difficulty in supporting the refinement category in their endeavor to obtain animal ethics with chronic pain investigations which in their very nature intentionally inflict persistent pain. Consequently, as the high economic and social stress of exaggerated female pain becomes more apparent, and the interest and need to research the etiology and management of chronic pain becomes more demanding (Casey and Dubner, 1989), there is an evident need to develop animal pain models that not only better reflect the heterogeneity of clinical chronic pain, but that are also capable of reducing the extent of animal suffering.

One of the most established chronic pain models is the CCI of the sciatic nerve model developed by Bennett and Xie (1988). Despite the wide application of this rodent pain method within the preclinical chronic pain literature, one key limitation is the binary all-or-none nature of the model. That is, the typical experimental design consists of a sham control that displays minimal alteration in mechanical allodynia, and the four chromic gut suture CCI group that display statistically significant and often maximal responses on a behavioral measure such as the von Frey test. Considering human chronic pain is derived from heterogeneous injuries, ranging from mild to severe, a model that deliberately generates only marked allodynia in itself is not a true representation of clinical pain (Grace et al., 2010) and is also unable to detect heterogeneity in the pain response. However, the recent development of a modified CCI model has been shown to successfully produce graded allodynia in male rodents, through variation in the number of sutures tied around the sciatic nerve (Grace et al., 2010). This innovative chronic pain model developed by Grace and colleagues not only better replicates the heterogeneous magnitude of triggers of chronic pain, but is able to detect a heterogenous pain response owing to differing degrees of pathology, that may provide further investigations a new and improved means to preclinically investigate sex differences in pain sensitivity without having to inflict maximal allodynia.

This study aimed to investigate for the first time the role of sex and oestrus cycle in a graded sciatic CCI pain model that produces heterogeneous degrees of mechanical allodynia. Additionally, the von Frey testing approach employed to assess mechanical allodynia was also examined combined with trialing of different statistical analysis approaches.

## Methods

### Subjects

Pathogen-free adult male and female Sprague-Dawley rats (300–350 g; University of Adelaide, Laboratory Animal Services, Waite Campus, Urrbrae, Australia) were utilized in all experiments in this study. Rats were housed in temperature- (18–21°C) and light-controlled (12 h light/ dark cycle; lights on at 07:00 h) rooms where standard rodent food and water was available *ad-libitum*. Preceding experimentation, rats were habituated to the animal holding care facility for 1 week, followed by 1 week of extensive experimenter handling and acclimatization to the von Frey testing apparatus in order to reduce successive handling stress. All procedures were approved by the Animal Ethics Committee of the University of Adelaide and were performed in accordance with the NHMRC Australian code of practice for the care and use of animals for scientific purposes and adhered to the guidelines of the Committee for Research and Ethical Issues of the IASP.

### Groups and design

This study utilized a novel graded sciatic nerve injury model of allodynia (Grace et al., 2010), a modified CCI model in the rat (Bennett and Xie, 1988), where 0, 1, 3, or 4 chromic gut sutures were placed around the sciatic nerve (N), to develop graded behavioral allodynia (varying degrees of allodynia) as described in detail previously (Grace et al., 2010). To ensure the systemic chromic gut challenge was equivalent between animals (Maves et al., 1993), additional equivalent chromic gut lengths were placed subcutaneously (S) over the hip, enabling each treated animal to be exposed to a total of four equivalent chromic gut pieces. Consequently, the treatment groups included N0S0, N0S4, N1S3, N3S1, and N4S0 animals, with eight males and eight females in each treatment group. Male and female rodents were followed to postoperative (PO) day 21 to determine whether the extent of nerve injury (number of chromic gut pieces around the sciatic nerve) modified the degree of allodynia.

### Pre-surgical and test procedures

Prior to CCI surgery and behavioral testing, a vaginal smear was taken from female rats using the common pipette smear technique to determine oestrus cycle phase (Marcondes et al., 2002). Smears were taken from eight females per phase, with results generated from a minimum of four consecutive oestrus cycles, with males “matched” according to assigned animal number and tested on the corresponding female test day. Vaginal smears were taken between 0800 and 1000 each testing and surgical day to minimize the incidence of transitional or “missed” stages (Sahar et al., 1997; Goldman et al., 2007). The four stages of the oestrous cycle pro-oestrus (pro), oestrus (oest), metoestrus (met), and dioestrus (di) could be recognized by the presence, absence or proportion of epithelial, cornified, and leucocyte cells (Goldman et al., 2007). In an attempt to replicate the small amount of stress experienced by female rats during the smear procedure, male rats were given a parallel injection of 1 ml/kg isotonic saline intraperitoneally.

### Chronic constriction injury surgery

The CCI model of chronic pain was performed at the mid-thigh level of the left hindleg as previously described (Grace et al., 2010). Rats were anaesthetized with isoflurane (3% in oxygen), fur shaved over the left mid-thigh and the skin cleaned. The sciatic nerve was aseptically exposed and isolated at mid-thigh level. One, three, or four loose chromic gut suture ligatures (cuticular 4-0 chromic gut, FS-2; Ethicon, Somerville, NJ, USA) were placed around the sciatic and once the superficial muscle overlying the nerve was sutured, additional chromic gut was placed subcutaneously. Whilst rescue opioid analgesia was on hand to administer to animals following surgery if an adverse event occurred, no such additional analgesia was provided following surgery, as commonly employed opioids have recently been demonstrated to exacerbate nerve injury-induced mechanical hypersensitivity (Watkins et al., 2009). Animals were monitored postoperatively until fully ambulatory prior to return to their homecage and checked daily for any sign of infection. No such cases occurred in this study.

### Allodynia behavior assessment

Throughout the study, testing was performed blind with regard to group assignment and oestrus cycle phase. Three diverse von Frey approaches were assessed in both male and female rats. All animals were examined using each von Frey technique, with each test separated by at least 2 h. Each von Frey test examined mechanical allodynia utilizing von Frey filaments across a range of thresholds in a sustained or phasic fashion. von Frey analyses examined in this study included: the classical 8-S method (Milligan et al., 2000) and Colburn method (Colburn et al., 1997) (henceforward termed Tests 1 and 2 respectively) introduced in detail below, in addition to a different von Frey approach, which combined aspects of Tests 1 and 2 using lower threshold stimuli, henceforth termed Test 3. For all von Frey techniques, testing was performed within the sciatic innervation region of the hindpaws as previously described in detail (Chacur et al., 2001; Milligan et al., 2001). Allodynia was characterized in all three behavioral tests as an intense paw withdrawal or licking of the stimulated hind paw. Assessments were made before surgery (baseline) and on postoperative days 3, 7, 10, 14, 17, and 21.

#### 8-S von Frey method (Test 1)

This test was performed as described previously (Milligan et al., 2000). Briefly, a logarithmic series of 10 calibrated Semmes-Weinstein monofilaments (von Frey filaments; Stoelting, Wood Dale, IL, USA) were applied for 8 s randomly to the left and right hindpaws of all animals in order to characterize the threshold stimulus intensity necessary to produce a paw withdrawal response. Log stiffness of the filaments was determined by log_10_ (milligrams × 10) and ranged from manufacturer designated 2.83 (0.07 g) to 5.18 (15.136 g) filaments. Behavioral responses were used to calculate absolute threshold (the 50% paw withdrawal threshold) by fitting a Gaussian integral psychometric function using a maximum-likelihood fitting method (Harvey, 1997; Treutwein and Strasburger, 1999) as described previously by Milligan et al. (2000, 2001).

#### Colburn von Frey method (Test 2)

Mechanical allodynia was also assessed utilizing a von Frey test which employed phasic application of stimulus at 2 point estimates along the rodent von Frey logarithmic force scale (the Colburn method; Test 2) (Colburn et al., 1997). Following previous pain investigations, all rodents were assessed for mechanical allodynia utilizing a 2 and 12 g von Frey filament (von Frey filaments; Stoelting, Wood Dale, IL, USA), which were applied within the sciatic innervation region of the left and right hind paws. Rats were subjected to a series of three sets of ten stimulations per filament, with filaments applied at 1 s intervals. 10 min break was provided in between each set of stimulations to avoid sensitization (Tanga et al., 2004). Behavioral responses were recorded as the average number of responses out of 30 for either the 2 or 12 g stimulus.

#### von Frey Test 3

By combining aspects of von Frey Tests 1 and 2, von Frey Test 3 investigated mechanical allodynia using phasic stimulation of von Frey filaments across a range of thresholds, including lower threshold filaments than those usually examined. Briefly, rats were subjected to 10 stimulations with 6 calibrated von Frey filaments (2.83, 0.07; 3.61, 0.40; 4.08, 1; 4.31, 2; 4.74, 6; 5.07, 10 g), chosen from the series of 10 utilized in von Frey Test 1. von Frey filaments were applied for 1 s at 1 s intervals. Filaments were not applied in ascending order of force, but rather random assignment each test session. In order to avoid sensitization, 10 min break was given between each set of stimulations, with 10 stimulations per filament. von Frey Test 3 investigated the response frequency at each von Frey filament and behavioral responses were recorded as the average number of responses out of 10 for each von Frey stimulus.

Results for all three von Frey tests that are provided following nerve injury are at the timepoint where allodynia was demonstrated stable (days 17–21).

### Statistics

For each of the von Frey tests, 1, 2, and 3, the relationship between the percent response and the variables sex, oestrus cycle phase, von Frey filament stimulus and surgery was assessed using linear modeling fitted using the statistical package R (R Development Core Team, 2011) via the graphical user interface: Rstudio (RStudio).

Initially a linear model was fitted that aimed to predict the percent response (number of positive responses out of a maximum of 30 (von Frey Test 2) or 10 (von Frey Test 3) by using the predictors sex, oestrus cycle phase, von Frey filament stimulus and surgery. The goal was to estimate the average increase in percent response for each increase in von Frey filament stiffness. We also wanted to estimate how this average increase is influenced by the various levels of sex, oestous cycle phase and surgery. As well, a modified linear modeling method was used, called mixed effect linear models, that had an extra term to account for the fact that we had repeated measures on the rats. This method allows us to account for the variation seen in the percent response both within and between the rats. Believing that there was a possibility for the influence of surgery and oestrous to depend on the rat's sex, we also added two terms to account for these interactions. The initial model (M1) was, therefore,


response ~ sex + oestrus + surgery + von
Frey stiffness + sex:oestrus + sex:surgery
+ (1|rat)


where response is the response rate expressed as a percentage (% response), sex is male or female, oestrus is pro-oestrus, oestrus, metoestrus or dioestrus, surgery is N0S0, N0S4, N1S3, N3S1, N4S0, von Frey stiffness is 1–6 and rat is the unique rat ID.

The model was fitted using the lmer() function from the lme4 package in R. The code is available on request from the authors.

An example R input would appear as:


M1 <- lmer(response ~ sex + oestrus +
surgery + von-Frey stiffness + sex:oestrus
+ sex:surgery + (1|rat), data=data)
summary(M1)


To ensure the simplest model to predict the percent response, the initial model (M1) was tested to assess if any for the predictors could be removed as there are not statistically significant predictors. The ability of the model to predict the percent response was measured using the Akaike's Information Criterion (AIC). The AIC measures how well the model fits the observations with a penalty term for the number of terms in the model. The penalty term is to try and ensure the most parsimonious model. Each predictor was removed from the model and the AIC measured to see if this caused a change in the AIC. The model with the smallest AIC was chosen. This process is repeated until the simplest model that predicts the percent response the best is obtained. This process is automated by the stepAIC() function from the MASS package (Venables and Ripley, 2002). This procedure indicated that none of the predictors could be removed without reducing the predictive ability of the model.

There is controversy regarding *P*-values for mixed effects models (Bates, 2006) and thus, we report observed *t*-values rather than *P*-values and use an observed *t*-value with absolute value of less than negative two or greater than two to indicate statistical significance. Owing to the statistical power of the model statistically significant but behaviorally small differences can be identified. As such, we only report statistical differences that are behaviorally relevant, representing for Test 2 a difference in percentage response of greater than 3.3, and 1.6% for von Frey test 3, as this represents a change in 1 response out of 10 on the tests.

To further elucidate differences in allodynia (percentage response) and each of the predictors (sex, oestrous cycle phase, surgery and von Frey filament stimulus), subsets of the data were considered (e.g., males only) and models fitted that predicted percent response for each of the predictors individually. Again the model used accounted for the repeated measures obtain from each rat.

An example R input of such subsets to investigate certain predictors:

Example 1. In order to investigate the effect of the female rodent oestrus cycle, male rodent data was excluded from the analysis. Below if the example R input in order to investigate this predictor individually:


# Model data excluding males: specifically
investigating the effect of the oestrus
cycle predictor:



M1<- lmer(response~(von Frey stiffness/
surgery/sex/(oestrus)+ (1|rat), data=subset
(data, sex==’female’))
summary(M1)


where response is the response rate expressed as a percentage (% response), sex is female, oestrus is pro-oestrus, oestrus, metoestrus or dioestrus, surgery is N0S0, N0S4, N1S3, N3S1, N4S0, von Frey stiffness is 1–6 and rat is the unique rat ID.

von Frey Test 2 was also examined using two different approaches. For each predictor (sex, oestrous cycle phase, surgery and von Frey filament stimulus), the difference between the allodynia (percentage response for the 2 g von Frey filament) for each level of the predictor was estimated. This was also repeated for the 12 g von Frey filament. Alternatively, an analysis model for covariance approach was utilized to assess the difference in intercept and slopes for linear regression lines fitted with allodynia (percentage response) as the response variable and von Frey filament stiffness as the predictor, with difference regression lines for each level of the fixed effect. The different approaches are equivalent but give difference contexts to compare the levels of the fixed effects.

## Results

### Experiment 1: importance of von Frey approach: choice of von Frey test determines sex difference at baseline

**Figure 1:**
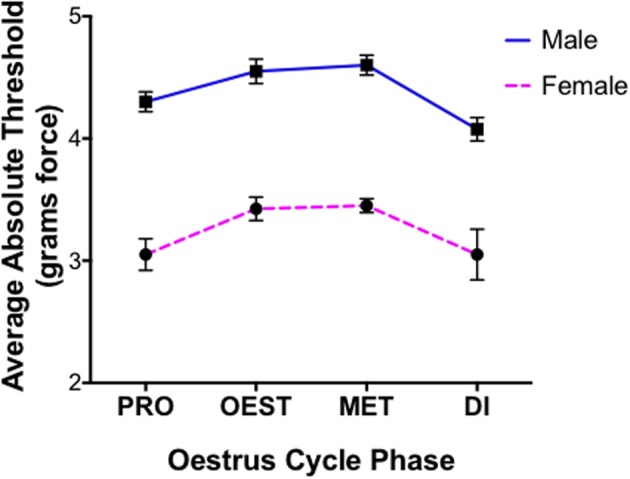
**Inability of von Frey method 1 to replicate healthy human pain thresholds**. Prior to nerve injury female mechanical allodynia was unable to be appropriately assessed utilizing the common 8-S method (von Frey Test 1). Results demonstrated the inability to distinguish female paw withdrawals indicative of allodynia, or those arising from repeated filament stimulation. Consequently, female rats displayed exceptionally low absolute thresholds compared to males across oestrus cycle phases prior to nerve injury (*t* = 14), contrasting healthy human pain thresholds. Assessments were made prior to CCI and behavioral responses were used to calculate absolute threshold (the 50% paw withdrawal threshold) by fitting a Gaussian integral psychometric function using a maximum-likelihood fitting method. A *t*-value of < −2 or >2 was determined statistically significant (*p* < 0.05). *n* = 8 per oestrus phase, per sex. Di, dioestrus; Met, metoestrus; Oest, oestrus; Pro, pro-oestrus.

Statistical differences for covariates generated utilizing von Frey Test 1 are expressed as the average difference in absolute threshold (the 50% paw withdrawal threshold). Investigating the role of sex in the graded nerve injury model revealed the inability of this von Frey test to distinguish between a female rodent paw withdrawal that was indicative of allodynia, or a paw withdrawal arising from repeated filament stimulation. As a consequence, females tested utilizing von Frey Test 1 were found to respond at the lowest von Frey filament examined (0.04 g force) and displayed extremely low absolute thresholds prior to nerve injury, responding significantly more than males prior to nerve injury (*t* = 14.0) (Figure 1). Accordingly, this von Frey technique failed to differentiate between factual observation (positive paw withdrawal) and the inference (pain) and as a consequence females were inappropriately deemed in pain, which did not resemble the factual scenario at baseline. In light of the inability to appropriately determine a female paw withdrawal response, this sex difference was thereby rendered meaningless and further results obtained using this von Frey approach are subsequently excluded from discussion.

### Experiment 2: von Frey Test 2 displays no sex differences at baseline, replicating healthy human pain sex differences

Mechanical allodynia was examined using von Frey Test 2 for the first time in a graded allodynia preclinical animal model. This study followed the traditional statistical approach whereby responses generated were independently examined at the 2 and 12 g von Frey filaments (DeLeo and Rutkowski, 2000; Sweitzer et al., 2001; LaCroix-Fralish et al., 2005a,b, 2006b). Results generated at individual von Frey filaments are expressed as the average difference in percent response.

Prior to nerve injury, baseline responses generated by von Frey Test 2 reflected healthy male and female nociceptive thresholds, displaying no significant difference between the sexes (Sarlani and Greenspan, 2002; Sarlani et al., 2004). Matched male and female rodents were found to respond equivalently at both the 2 and 12 g von Frey filaments prior to nerve injury (2 g, *t* = 0.20; 12 g, *t* = 0.43; Table 1), with the average difference in percent response failing to reach behavioral significance (2 g: 0.21%; 12 g: 0.42%; Table 1). Prior to nerve injury, this testing method further revealed the absence of an oestrus cycle effect. At baseline, female responses were unaffected by the rodent oestrus cycle, with an average percent difference in response of only 0.40% between phases at both the 2 g (*t* ≥ −2 but ≤1.7, Table 2) and 12 g (*t* ≥ −2 but ≤0.27, Table 3) von Frey filaments.

**Table 1:** **Traditional statistical analysis at independent von Frey Filaments for von Frey Test 2 revealed equivalent male and female baseline (Pre-Surgery) responses**.

	**2 g Male (% response)**	**2 g Female (% response)**	**2 g Difference in percent response (%) male:female**	**2 g Is the sex difference significant? (*t* =) male:female**	**12 g Male (% response)**	**12 g Female (% response)**	**12 g Difference in percent response (%) male:female**	**12 g Is the sex difference significant? (*t* =) male:female**
Pre-surgery	3.8 ± 0.12	4.0 ± 0.18	0.21	0.20	9.0 ± 0.18	9.2 ± 0.14	−0.42	−0.43
N0S0	4.6 ± 0.23	4.8 ± 0.19	0.21	0.20	9.2 ± 0.12	10.0 ± 0.17	−0.42	−0.43
N0S4	9.4 ± 0.21	12.0 ± 0.24	−2.5	−1.6	11.0 ± 0.24	18.0 ± 0.22	−3.5	−2.3
N1S3	14.0 ± 0.31	17.0 ± 0.30	−2.9	−2.7	26.0 ± 0.33	32.0 ± 0.32	**−6.5**	**−6.3**
N3S1	27.0 ± 0.31	41.0 ± 0.31	**−14.0**	**−7.3**	38.0 ± 0.22	60.0 ± 0.24	**−22.0**	**−11.0**
N4S0	36.0 ± 0.38	51.0 ± 0.42	**−15.0**	**−8.1**	52.0 ± 0.31	78.0 ± 0.38	**−27.0**	**−13.0**

Once allodynia became stable (days 17–21) N4S0 and N3S1 females were significantly more allodynic than matched males however, a significant sex difference was not confirmed for N1S3 rodents, failing to demonstrate this sex effect using the 2 g von Frey filament. Results generated at individual von Frey filaments are expressed as the average difference in percent response, generated from the response rates (the percent response /30; Tables A1, A2). A t-value of < −2 or >2 was determined statistically significant (p < 0.05) when difference in percentage response were greater than 3.3%. n = 8 per treatment group, per sex. N, number of sciatic sutures, S, number of subcutaneous sutures; g, grams force.

**Table 2:** **Traditional statistical analysis at the independent 2 g von Frey Filament for von Frey Test 2 revealed the absence of an oestrus cycle effect prior to nerve injury (Pre-Surgery)**.

	**PRO:OEST**		**PRO:MET**		**PRO:DI**		**OEST:MET**		**DI:OEST**		**DI:MET**	
	**%**	***t* =**	**%**	***t* =**	**%**	***t* =**	**%**	***t* =**	**%**	***t* =**	**%**	***t* =**
Pre-surgery	−0.77	−0.19	−0.60	−0.58	−0.53	0.15	0.20	−0.39	−0.25	−0.34	0.051	1.7
N0S0	0.30	1.9	1.1	1.7	0.80	−0.8	0.80	1.7	−0.51	−0.83	0.30	1.5
N0S4	2.1	1.0	2.0	1.7	−1.1	1.1	−0.11	−1.3	3.2	0.86	3.1	0.67
N1S3	**5.1**	**6.7**	**5.8**	**6.3**	0.51	−0.60	0.27	−0.42	**7.6**	**6.7**	**7.3**	**6.3**
N3S1	1.2	0.97	1.2	1.0	−0.93	−0.57	−7.8	1.1	2.2	−1.3	2.2	0.87
N4S0	0.70	1.6	3.1	1.7	−6.0	−0.073	2.7	0.64	2.7	0.74	2.3	1.2

Once allodynia became stable (days 17–21) females (N1S3) in pro-oestrus and dioestrus displayed heightened mechanical sensitivity. Results generated at individual von Frey filaments are expressed as the average difference in percent response, generated from the response rates (the percent response /30; Table A1). A t-value of < −2 or >2 was determined statistically significant (p < 0.05) when difference in percentage response were greater than 3.3%. n = 8 per treatment group, per sex. N, number of sciatic sutures, S, number of subcutaneous sutures; g, grams force. %, the average difference in percent response; t =, is there a significant effect of oestrus cycle?

**Table 3:** **Traditional statistical analysis at the independent 12 g von Frey Filament for von Frey Test 2 revealed the absence of an oestrus cycle effect prior to nerve injury (Pre-Surgery)**.

	**PRO:OEST**		**PRO:MET**		**PRO:DI**		**OEST:MET**		**DI: OEST**		**DI:MET**	
	**%**	***t* =**	**%**	***t* =**	**%**	***t* =**	**%**	***t* =**	**%**	***t* =**	**%**	***t* =**
Pre-surgery	−1.7	−0.39	−1.3	−0.29	−1.1	0.27	0.44	−0.79	−0.48	−0.64	−0.10	−1.3
N0S0	0.9	0.42	0.44	1.2	0.48	−0.40	0.54	−0.71	0.77	0.32	1.0	1.3
N0S4	1.1	2.1	−0.11	1.7	−1.0	0.90	−1.6	−0.71	2.7	0.22	1.4	2.4
N1S3	2.4	2.2	**6.3**	**3.7**	0.25	1.2	−0.18	−0.24	2.6	1.9	2.0	3.1
N3S1	0.27	0.19	−2.7	2.1	−0.32	0.14	0.44	1.7	0.43	−0.83	0.43	2.2
N4S0	0.12	2.3	0.81	1.9	−1.4	0.024	0.50	0.92	1.2	2.1	2.1	2.1

An oestrus cycle effect could not be conclusively established following nerve injury with females responding significantly more throughout pro-oestrus, but only when compared to metoestrus and no other cycle phase. Results generated at individual von Frey filaments are expressed as the average difference in percent response, generated from the response rates (the percent response /30; Table A2). A t-value of < −2 or >2 was determined statistically significant (p < 0.05) when difference in percentage response were greater than 3.3%. n = 8 per treatment group, per sex. n = 8 per oestrus phase. N, number of sciatic sutures; S, number of subcutaneous sutures; g, grams force. Di, dioestrus; Met, metoestrus; Oest, oestrus; Pro, pro-oestrus. %, the average difference in percent response; t =, is there a significant effect of oestrus cycle?

### von Frey Test 2 reveals graded chronic pain in the female rat

Application of von Frey Test 2 and statistical analysis at the independent 2 and 12 gram von Frey filaments demonstrated graded chronic pain for the first time in both the male and female rat. Increasing the number of sciatic sutures significantly increased the number of responses indicative of allodynia for both sexes (2 g: *t* ≤ −4.4; 12 g: *t* ≤ −11.0, Table 1). Furthermore, chromic gut itself was demonstrated to cause allodynia, with N0S4 males and females having significantly greater allodynia compared to N0S0 rats (*t* ≤ −2.6, Table 1). Consequently, examining responses at independent von Frey filaments replicated previous findings of a graded chronic pain model in the male rodent and demonstrated an allodynia dose-response relationship for the first time in females.

### Assessment of mechanical hypersensitivity at individual von Frey filaments fails to appropriately investigate the role of sex in allodynia

Sex differences in mechanical allodynia were subsequently examined in the graded chronic pain model utilizing von Frey Test 2. When allodynia became stable, females (N4S0) were significantly more allodynic than matched males (2 g: 15.0%, *t* = −8.1; 12 g: 27.0%, *t* = −13.0, Table 1), replicating previous studies that have utilized the traditional CCI four-suture approach. This study further demonstrated for the first time a sex difference in moderately allodynic animals, with N3S1 females responding significantly more than matched N3S1 males (2 g: 14.0% *t* = −7.3; 12 g: 22.0%, *t* = −11.0, Table 1). This sex difference, however, was not conclusively established in N1S3 rodents, failing to refine preclinical chronic pain testing. Although N1S3 females were found significantly more allodynic than matched males when tested utilizing the 12 g von Frey filament (6.5%, *t* = −6.3, Table 1), this sex difference was not replicated with the 2 g filament (2.9%, *t* = −2.7, Table 1).

### Independent filament analysis fails to determine an oestrus cycle effect

Traditional statistical analysis at independent von Frey filaments demonstrated the inability for von Frey Test 2 to conclusively demonstrate an oestrus cycle effect on female mechanical allodynia following nerve injury. When allodynia became stable, females (N1S3) displayed heightened mechanical sensitivity throughout both dioestrus and pro-oestrus (2 g: ≥ 5.1%, *t* ≥ 6.3, Table 2). These results however, were not replicated using the 12 g von Frey filament, where females responded significantly more throughout pro-oestrus, but only when compared to metoestrus and no other cycle phase (Pro:Met, 6.3%, *t* = 3.7, Table 3).

### Investigating the role of sex and the oestrus cycle using von Frey Method 2: examining differences across von Frey filaments

Considering the inability of von Frey Test 2 to conclusively establish both sex and oestrus cycle differences using the traditional statistical approach whereby mechanical allodynia is assessed at independent von Frey filaments, this study examined an alternative statistical approach whereby the relationship between the two point estimates was examined (slope). Considering the ability to accurately estimate a difference in slope utilizing data from upper and lower limits of a scale, this study investigated the estimate of the slope of the linear relationship between the response and von Frey filament. Results are expressed as the percent difference in slope between the response and two von Frey filaments.

Following an examination of a difference in slope, von Frey Test 2 revealed baseline rodent allodynia scores reflected healthy human pain thresholds (Sarlani and Greenspan, 2002; Sarlani et al., 2004), with males and females responding equivalently prior to nerve injury (male:female, −0.25%, *t* = −0.71, Table 4). Moreover, prior to nerve injury, the rodent oestrus cycle also failed to influence female allodynia scores, with less than a 1% difference in slope between phases, replicating the findings at both the 2 and 12 g von Frey filaments (Section Experiment 2: von Frey Test 2 Displays No Sex Differences at Baseline, Replicating Healthy Human Pain Sex Differences) (*t* ≥ −1.1 but ≤1.4, Table 5).

**Table 4:** **Responses generated from von Frey Test 2 were further used to examine the estimate of the slope of the linear relationship between the response and von Frey filament**.

	**Male (% response)**	**Female (% response)**	**Difference in slope (%) male:female**	**Is the sex difference significant? (*t* =) male:female**
Pre-surgery	6.4	6.6	−0.25	−0.71
N0S0	7.1	7.2	−0.10	−0.12
N0S4	12.0	15.0	−3.0	−2.1
N1S3	20.0	21.0	−1.0	−1.7
N3S1	33.0	50.0	**−18.0**	**−7.5**
N4S0	44.0	65.0	**−21.0**	**−6.9**

This alternative statistical analysis revealed equivalent male and female responses prior to nerve injury (Pre-Surgery) however a sex difference was confirmed once allodynia became stable (days 17–21) where N3S1 and N4S0 females were found significantly ore allodynic than matched males. Results are expressed as the percent difference in slope between the response and two von Frey filaments, generated from response rates (the percent response /30 across von Frey filaments; Table A3). A t-value of < −2 or > 2 was determined statistically significant (p < 0.05) when difference in percentage response were greater than 3.3%. n = 8 per treatment group, per sex. N, number of sciatic sutures; S, number of subcutaneous sutures; g, grams force.

**Table 5:** **Examining the estimate of the slope of the linear relationship between the response and von Frey filament for von Frey Test 2 revealed the absence of an oestrus cycle effect across all surgery groups**.

	**PRO:OEST**		**PRO:MET**		**PRO:DI**		**OEST:MET**		**DI: OEST**		**DI:MET**	
	**%**	***t* =**	**%**	***t* =**	**%**	***t* =**	**%**	***t* =**	**%**	***t* =**	**%**	***t* =**
Pre-Surgery	−0.44	1.4	−0.38	−1.1	−0.018	−0.050	0.059	0.19	0.29	1.2	−0.37	1.1
N0S0	0.42	0.29	−0.56	−0.51	−1.3	−1.1	−0.11	−0.10	1.7	−1.1	−1.8	−0.86
N0S4	2.4	1.1	0.10	−1.2	1.1	−0.19	−1.0	−0.86	0.11	−1.80	−0.91	−0.97
N1S3	−1.8	0.78	−1.9	−1.2	1.1	0.36	−0.21	−0.029	−2.9	−0.65	−3.1	−0.73
N3S1	−1.9	−0.22	−2.5	−0.43	−1.8	−0.31	−1.0	−0.11	−0.025	−0.0030	−1.08	0.79
N4S0	−1.1	−0.16	2.7	0.61	1.8	0.28	−3.0	0.71	−3.0	−0.40	0.012	0.32

Results are expressed as the percent difference in slope between the response and two von Frey filaments, generated from response rates (the percent response /30 across von Frey filaments; Table A3). A t-value of < −2 or >2 was determined statistically significant (p < 0.05) when difference in percentage response were greater than 3.3%. n = 8 per treatment group, per sex. n = 8 per oestrus phase. N, number of sciatic sutures; S, number of subcutaneous sutures; g, grams force. Di, dioestrus; Met, metoestrus; Oest, oestrus; Pro, pro-oestrus. %, the average difference in percent response; t =, is there a significant effect of oestrus cycle?

A graded allodynia response was also revealed when determining a slope difference between pain treatment groups following nerve injury. Increasing the number of sciatic sutures was demonstrated to significantly increase the number of responses indicative of allodynia for both sexes (≥11.0%, *t* ≤ −5.6, Table 4). This robust mechanical allodynia was observed in both male and female rats once allodynia became stable.

Post nerve injury, von Frey Test 2 demonstrated females were considerably more allodynic than males in the graded nerve injury model. Once allodynia became stable, a significant difference in slope was demonstrated between N4S0 (male:female, 21.0%, *t* = −6.9, Table 4) and N3S1 rats (male:female, 18.0%, *t* = −7.5, Table 4). This sex difference however, was not demonstrated between N1S3 rodents (1.0%, *t* = −1.7, Table 4) and female rodent mechanical allodynia was further demonstrated unaffected by the oestrus cycle for all pain treatment groups (≥−3.1% but ≤3.0%, *t* ≥ −1.8 but ≤1.1; Table 5).

### Experiment 3: von Frey Test 3: a behavioral technique to investigate the role of sex and the rodent oestrus cycle in mechanical hypersensitivity

von Frey Test 3 examined the relationship between the response and the force of the von Frey filament. Results generated by von Frey Test 3 are expressed as the percent difference in response per increase in von Frey hair stiffness.

**Figure 2:**
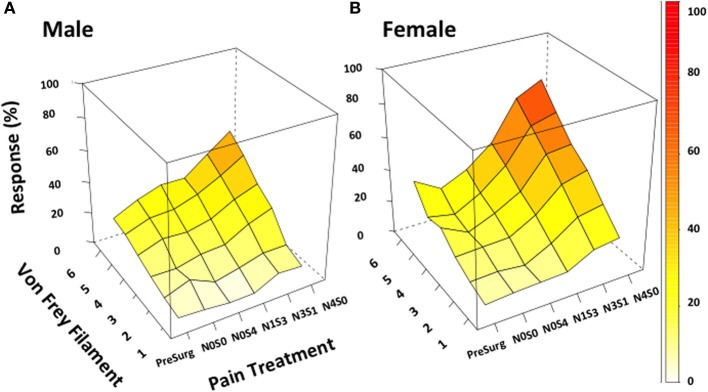
**von Frey test 3 demonstrates the ability to investigate chronic pain mechanisms without inflicting maximal pain**. Appropriately investigating the role of sex in chronic pain became possible utilizing von Frey Test 3. Using this adapted behavioral test this study demonstrated the successful generation of a graded chronic pain model in both the male and female rat. Increasing the number of sciatic sutures was found to significantly increase the percentage response rate per von Frey filament in both males **(A)** and females **(B)** (≤−1.6%, *t* ≥ 4.9). Illustrating refinement of the traditional CCI procedure, females (N1S3, N3S1) were demonstrated statistically more allodynic than males without having to inflict maximal pain (*t* = −3.4) when investigating the difference in percent response per increase in von Frey filament stiffness. von Frey Test 3 data is presented as a 3D surface plot of the response rate out of 10, recorded as a percentage. 3D surface plots were generated using the R Studio lattice package. Results were analyzed utilizing repeated measures linear mixed effects modeling. A *t*-value of < −2 or >2 was determined statistically significant (*p* < 0.05). *n* = 8 per treatment group, per sex. von Frey filaments: 6: 10, 5: 6, 4: 2, 3: 0.6, 2: 0.4, 1: 0.07 g. N, number of sciatic sutures; S, number of subcutaneous sutures; g, grams force.

Utilizing von Frey Test 3, graded mechanical allodynia was established in both the male and female rat once allodynia became stable. Following nerve injury, increasing the number of sciatic sutures significantly increased the level of allodynia in both rodent sexes (≤−1.6%, *t* ≥ 4.9; Table 6, Figure 2). Of particular importance, a significant differentiation was established between N0S0 and N0S4 males and females specifically, where N0S4 rodents responded significantly more compared to N0S0 rats (≥2.3%, *t* ≥ 2.4, Table 6), indicating chromic gut itself causes allodynia.

**Table 6:** **von Frey Test 3 examined the relationship between the response and the force of the von Frey filament, across a range of filaments**.

	**Male (%response)**	**Female (%response)**	**Difference in response per increase in von Frey hair stiffness male:female**	**Is the sex difference significant? male:female**
Pre-surgery	3.9 ± 0.11	4.7 ± 0.10	−0.83	−1.1
N0S0	4.0 ± 0.23	4.9 ± 0.28	−0.64	−1.8
N0S4	6.3 ± 0.24	8.1 ± 0.30	0.80	−1.6
N1S3	6.4 ± 0.29	10.1 ± 0.30	**−3.6**	**−3.4**
N3S1	8.6 ± 0.32	12.0 ± 0.30	**−3.4**	**−7.7**
N4S0	12.1 ± 0.48	13.6 ± 0.50	−1.5	−1.7

Prior to nerve injury (Pre-Surgery), von Frey Test 3 determined equivalent male and female responses. Once allodynia became stable (days 17–21) a sex difference was established, with N1S3 and N3S1 females significantly more allodynic than matched males. Results generated by von Frey Test 3 are expressed as the percent difference in response per increase in von Frey hair stiffness, generated from the estimated increase in percent response per increase in von Frey stiffness; Table A4. A t-value of < −2 or >2 was determined statistically significant (p < 0.05) when differences in percentage response were greater than 1.6%. n = 8 per treatment group, per sex. N, number of sciatic sutures; S, number of subcutaneous sutures; g, grams force.

Prior to CCI, male and female rats examined using this von Frey testing method were found to respond equivalently, with an average difference in response per increase in von Frey hair stiffness of only 0.83% (Table 6, Figure 2), thereby replicating the clinical pain scenario. Female rodent mechanical allodynia was also demonstrated unaffected by the rodent oestrus cycle prior to nerve injury, with an average difference of merely 1% between phases (*t* ≥ −1.5 but ≤1.6, Table 7).

**Table 7:** **von Frey Test 3 examined the relationship between the response and the force of the von Frey filament, across a range of filaments**.

	**PRO:OEST**		**PRO:MET**		**PRO:DI**		**OEST:MET**		**DI: OEST**		**DI:MET**	
	**%**	***t* =**	**%**	***t* =**	**%**	***t* =**	**%**	***t* =**	**%**	***t* =**	**%**	***t* =**
Pre-Surgery	−0.83	−0.88	−1.43	−1.5	−0.95	−0.83	0.59	0.69	0.20	0.98	−0.4	1.6
N0S0	0.10	−0.68	−0.10	−1.2	0.20	−0.68	−0.20	0.45	1.1	−0.67	0.9	1.2
N0S4	−1.1	−1.1	−0.60	−0.63	−1.0	−0.34	0.50	0.60	−0.80	−0.94	−0.30	−0.40
N1S3	**1.7**	**2.2**	**2.1**	**2.4**	0.20	−0.020	0.40	0.49	**2.0**	**2.6**	**1.9**	**2.4**
N3S1	**2.1**	**3.5**	**2.9**	**3.4**	0.097	0.12	−0.80	−1.4	**2.1**	**3.3**	**3.0**	**3.0**
N4S0	1.0	1.3	1.0	0.39	1.0	0.74	0.010	−0.94	0.013	0.60	−0.012	−0.40

Prior to nerve injury (Pre-Surgery), the rodent oestrus cycle did not influence female rodent responses. Following nerve injury, once allodynia became stable (days 17–21) von Frey Test 3 revealed an oestrus cycle effect, with N1S3 and N3S1 females significantly more allodynic throughout pro-oestrus and dioestrus. Results generated by von Frey Test 3 are expressed as the percent difference in response per increase in von Frey hair stiffness, generated from the estimated increase in percent response per increase in von Frey stiffness; Table A4. A t-value of < −2 or >2 was determined statistically significant (p < 0.05) when differences in percentage response were greater than 1.6%. n = 8 per oestrus phase. N, number of sciatic sutures; S, number of subcutaneous sutures; g, grams force. Di, dioestrus; Met, metoestrus; Oest, oestrus; Pro, pro-oestrus. %, the average difference in percent response; t =, is there a significant effect of oestrus cycle?

**Figure 3:**
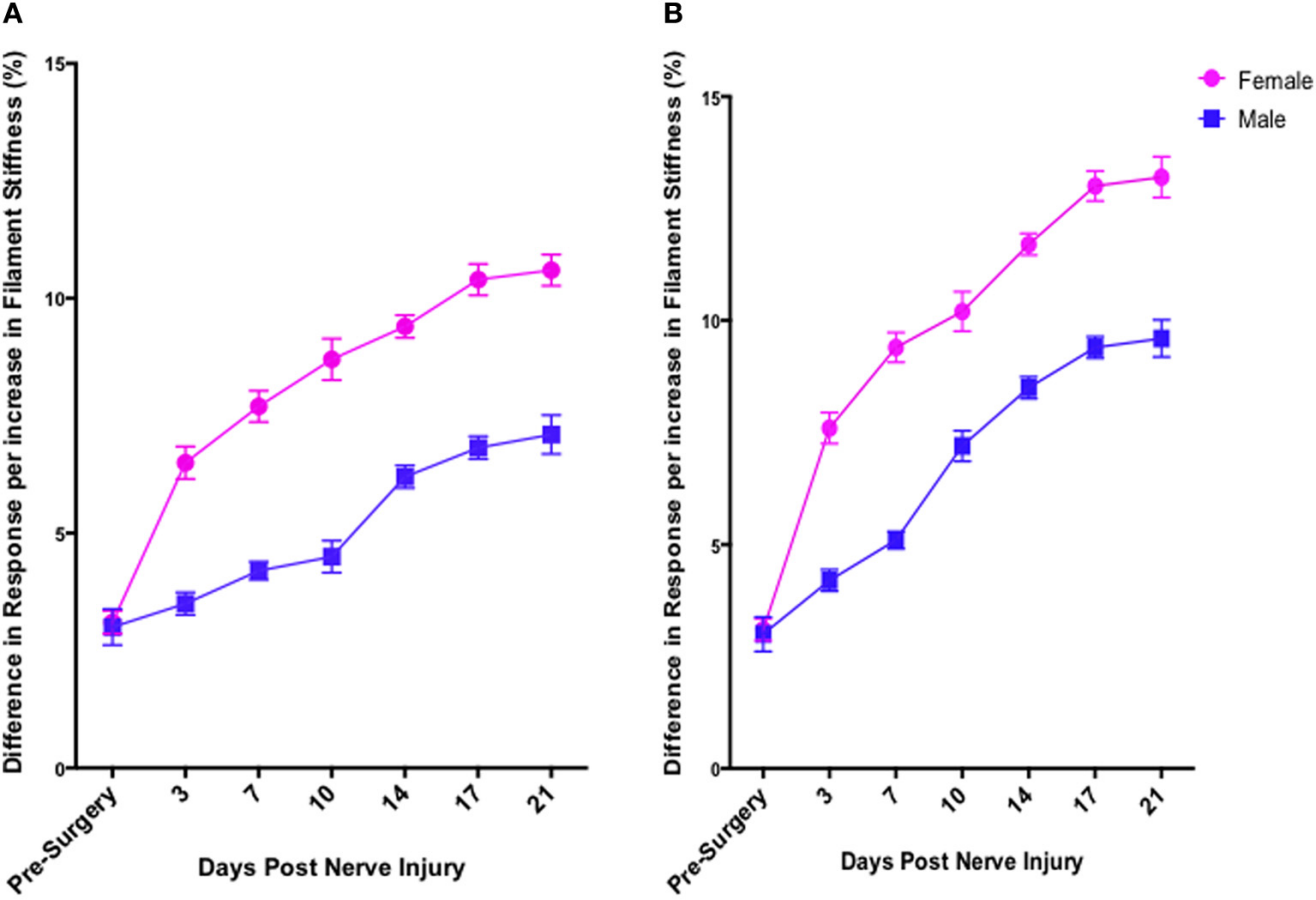
**von Frey test 3: a behavioral technique refining the investigation of the role of sex and rodent oestrus cycle in mechanical hypersensitivity**. Through to 21 days following nerve injury, von Frey Test 3 was the sole behavioral testing method examined that identified a contributing role of sex and oestrus cycle in female mechanical allodynia examined in the graded nerve injury model. N1S3 females were found significantly more allodynic than matched males **(A)** and the same was found for N3S1 female rodents **(B)**. Results generated by von Frey Test 3 are expressed as the percent difference in response per increase in von Frey hair stiffness. Results were analyzed utilizing repeated measures linear mixed effects modeling. A *t*-value of < −2 or >2 was determined statistically significant (*p* < 0.05). *n* = 8 per treatment group, per sex. N, number of sciatic sutures; S, number of subcutaneous sutures; g, grams force.

**Figure 4:**
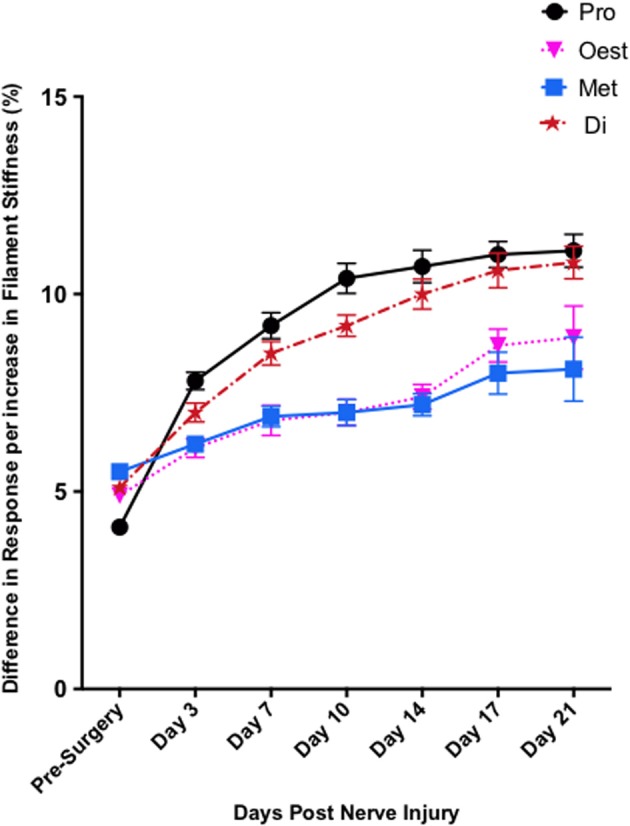
**Exacerbated female pain is influenced by the rodent oestrus cycle**. von Frey Test 3 demonstrated female mechanical allodynia was heavily dependent on the rodent oestrus cycle. Post nerve injury, moderate pain females (N3S1) were significantly more allodynic during dioestrus and pro-oestrus *t* = −7.7. The data presented is generated from von Frey Test 3. Results generated by von Frey Test 3 are expressed as the percent difference in response per increase in von Frey hair stiffness. Results were analyzed utilizing repeated measures linear mixed effects modeling. A *t*-value of < −2 or >2 was determined statistically significant (*p* < 0.05). *n* = 8 per treatment group. von Frey filaments: 6: 10, 5:6, 4: 2, 3: 1.0, 2: 0.4, 1: 0.07 g. Di, dioestrus; met, metoestrus; oest, oestrus; pro, pro-oestrus.

In contrast to Test 2, von Frey Test 3 not only revealed a sex difference in mechanical sensitivity following nerve injury but further revealed the ability of the novel sciatic nerve injury model to refine the traditional CCI technique (Figure 3). Once allodynia became stable, application of von Frey testing method 3 revealed exacerbated clinical female pain, with a significant difference in response per increase in von Frey hair stiffness between N1S3 and N3S1 males and females. For the first time, N1S3 females were found to respond 3.6 % more than matched males per von Frey filament (*t* = −3.4; Table 6, Figure 3A) and N3S1 females responding 3.4% more than matched males per von Frey filament (*t* = −7.7; Table 6, Figure 3B). von Frey Test 3 further revealed a hormonal influence on female mechanical hypersensitivity once allodynia became stable. Unlike Test 2, this von Frey testing method revealed exacerbated female mechanical allodynia throughout the phases of dioestrus and pro-oestrus in both N1S3 and N3S1 rodents (N1S3: ≥1.7%, *t* ≥ 2.2; N3S1: ≥2.1%, *t* ≥ 3.3, Table 7, Figure 4).

## Discussion

This study aimed to investigate for the first time the role of sex and oestrus cycle in a graded sciatic CCI pain model that produces heterogeneous degrees of mechanical allodynia. Additionally, the von Frey testing approach employed to assess mechanical allodynia was also examined combined with trialing of different statistical analysis approaches.

This aim was successfully achieved utilizing a modified von Frey testing approach, accompanied by unique statistical analysis. Here we demonstrated that testing the number of responses out of 10 tests across all 6 von Frey hairs in N1S3 sciatic injury was sufficient to observe significant sex and oestrous phase effects. In contrast, the other two approaches were not able to achieve this degree of behavioral sensitivity. Importantly, the use of the graded constriction injury identified that the current four suture model produces unnecessary maximal allodynia with similar conclusions able to be drawn employing only one suture.

In order to preclinically investigate the mechanisms underlying the female prevalence of chronic pain, studies need to employ models and behavioral testing methods that best replicate the clinical scenario. To examine sex differences in chronic pain utilizing the novel graded sciatic nerve injury model, this study investigated male and female mechanical nociception using three von Frey testing approaches. Despite numerous studies exploiting the technique of von Frey to investigate rodent mechanical hypersensitivity, this study revealed the profound differences in conclusions that can be drawn from the use of different von Frey procedures, as well as the importance of the statistical approach to investigate female rodent mechanical hypersensitivity.

Experiment 1 investigated the role of sex prior to nerve injury utilizing von Frey Test 1. This study demonstrated the inability of this test to appropriately infer female pain from a positive paw withdrawal given that females were erroneously found significantly more allodynic than males. Since adapted by Milligan et al. (2000), von Frey Test 1 has successfully investigated both the attenuation and the exacerbation of male mechanical hypersensitivity (Milligan et al., 2003, 2004; Ledeboer et al., 2005; Hutchinson et al., 2008, 2009, 2010; Grace et al., 2010; Loram et al., 2011). To our knowledge however, within the very few investigations that have utilized this particular von Frey test to examine sex differences in chronic pain, some have also reported non-sensically high female baseline mechanical thresholds (prior to any pharmacological, genetic or surgical intervention). Such was demonstrated in a study by Mogil and Colleagues investigating sex differences in mechanical allodynia in C57BL/6 mice utilizing a comparable von Frey technique (Mogil et al., 2006). In our study, observed higher baseline female activity misinterpreted by this particular application of von Frey as a pain response may potentially elucidate lower female mechanical thresholds compared with males. Interestingly, in a discussion of the issues associated with monofilament pain testing, Bove identifies that the application of sustained filament stimuli may in fact be examining different sensory modalities such as itch, rather than pain (Bove, 2006). Considering the already known translatability issues associated with preclinical animal pain studies, the continued use of behavioral paradigms that fail to appropriately infer pain casts further doubt on the validity of animal pain studies (Mogil, 2009). As a direct result, this study questions the validity of this method's use in future pain investigations examining female mechanical allodynia.

Although previously utilized by chronic pain investigations to examine rodent mechanical allodynia, this study employed von Frey Test 2 for the first time in a graded allodynia preclinical animal model to investigate the role of sex and the rodent oestrus cycle. Following the traditional statistical approach to this method whereby allodynia was examined at two independent von Frey filaments, a large degree of variability between von Frey hairs led to the need for an alternative statistical analysis. Although the majority of chronic pain investigations conventionally analyse mechanical allodynia using a logistic equation (using essential features of the stimulus response function such as the maximal response, the response threshold and the 50% of maximal response), data generated from von Frey Test 2 at only two points along the von Frey logarithmic force scale did not permit this conventional statistical analysis (owing to the lack of data). Rather the estimates generated from upper and lower limits of this scale enabled the capacity to accurately estimate the slope between response and von Frey filament. In doing so, this unconventional statistical approach provided an improved means to investigate the relationship between the sexes and oestrus cycle phases. To our knowledge this study is the first to examine a difference in slope in rodent mechanical allodynia. Using this alternative statistical approach, significantly greater female mechanical hypersensitivity was demonstrated in N3S1 recipients post neuropathic injury, replicating previous findings in an array of animal chronic pain models (Coyle et al., 1995; DeLeo and Rutkowski, 2000; LaCroix-Fralish et al., 2005a,b; Li et al., 2009). Despite bypassing the large degree of inconsistency associated with a point estimate approach, examining a difference in slope failed to demonstrate well-founded sex differences in N1S3 rats as well as an oestrus cycle effect in all pain treatment groups post neuropathic injury.

In the interest of continuing preclinical animal pain research the global aim of this study was to investigate male and female mechanical nociception in a model capable of reducing animal suffering compared to existing models. As demonstrated in Experiment 3, von Frey Test 3 was the only testing method to reveal the ability of the novel sciatic nerve injury model to refine the traditional CCI technique, firmly establishing sex and oestrus cycle differences in less allodynic rodents (N1S3 subjects). In order to compare the findings between von Frey Tests 2 and 3, and distinguish where von Frey Test 3 in fact identified sex and oestrus cycle differences that Test 2 was unable to detect, this study unconventionally examined the relationship (slope) between the response and the force of the von Frey filament for responses generated by von Frey Test 3. As previously mentioned, historically, chronic pain investigations conventionally use a logistic approach (reporting maximal response, the response threshold and the 50% of maximal response). Although von Frey Test 3 could have been statistically examined conventionally, and may be examined as such in future investigations, this was not possible with von Frey Test 2 (see above). Consequently, in order to make comparisons between the two von Frey methods, von Frey Test 3 investigated the relationship between the response and the force of the von Frey filament (the slope).

The inability of von Frey Method 2 to refine preclinical chronic pain testing, failing to identify sex and oestrus cycle differences in less allodync animals, upon further analysis came as a result of testing at the high end of the von Frey logarithmic force scale. Parameterization of results generated by von Frey Test 3 revealed the particular von Frey filaments at which sex and oestrus cycle where statistical differences occurred, with numerous filaments at the low end of the von Frey logarithmic force scale found to contribute to the overall statistical effects of this von Frey test (Table 8). The ability to assess the effect of covariates on the allodynia response for a range of von Frey filaments is not achievable using von Frey Test 2, where differences can only be assessed at the two point estimates or by inferring differences for von Frey filaments between these values on interpolation based on the assumption of linearity. Consequently, parameterization revealed the advantages of testing over a range of von Frey filaments, most importantly lower threshold filaments when examining female rodent mechanical sensitivity, emphasizing the effectiveness of von Frey Test 3.

**Table 8:** **The ability of von Frey test 3 to estimate differences in allodynia for diverse covariates across individual von Frey filaments**.

**von Frey filament (grams force)**	**Statistical sex effect (*t* < −2 or >2) N1S3 subjects**	**Statistical sex effect (*t* < −2 or >2) N3S1 subjects**	**Statistical oestrus effect (*t* < −2 or >2) N3S1 subjects**	**Statistical graded surgical effect (*t* < −2 or >2)**
Filament 1 (0.07)	✓	✓	✖	✖
Filament 2 (0.4)	✓	✓	✓	✖
Filament 3 (1.0)	✓	✓	✓	✓
Filament 4 (2.0)	✓	✓	✓	✓
Filament 5 (6.0)	✓	✓	✓	✓
Filament 6 (10.0)	✓	✓	✓	✓

Parameterization of repeated measures linear mixed effects modeling exposed the specific von Frey filaments at which the adapted von Frey Test 3 demonstrated sex, oestrus cycle and surgical statistical differences. The modified von Frey technique demonstrated the successful generation of a graded chronic pain model by which sex differences were established for the first time in rodents with reduced pain, and the rodent oestrus cycle found to undoubtedly influence female pain (Section Experiment 3: von Frey Test 3: A Behavioral Technique to Investigate the Role of Sex and the Rodent Oestrus Cycle in Mechanical Hypersensitivity). The sole ability of this von Frey test to indisputably demonstrate such effects resulted from testing over a range of von Frey filaments. Parameterization revealed statistical differences at the low end of the von Frey logarithmic force scale, thereby demonstrating their contribution to the positive findings utilizing this method. The data presented is generated from von Frey Test 3. Results were analyzed utilizing repeated measures linear mixed effects modeling, where the repeated measures of the experimental design was also reparameterized using the contrast() function in the contrast R package (Kuhn, 2011) and the glht() function in the multcomp R package (Hothorn et al., 2008). This enabled the assessment of the difference in the percentage responses for different levels of each fixed effect, sex, oestrous, surgery, for different weights of von Frey filaments. A t-value of < −2 or >2 was determined statistically significant (p < 0.05). To demonstrate the von Frey filaments at which statistical differences occured, ✓-indicate a positive finding, whereas ✖-signify the failure to determine a significant effect. n = 8 per treatment group, per sex. Smears were taken from eight females per phase, with results generated from a minimum of four consecutive oestrus cycles. von Frey filaments: 2.83: 0.07, 3.61: 0.4, 4.08: 1, 4.31: 2, 4.74: 6, 5.07: 10.

It is hypothesized that poorly translatable preclinical methodologies are partly responsible for the incomplete understanding of exacerbated female pain. Therefore, chronic pain investigations need to utilize methods which best replicate the clinical pain scenario. Although studies examining sex differences in mechanical pain thresholds have yielded variable findings, investigations using phasic noxious stimuli consistently demonstrate equivalent healthy male and female thresholds (Sarlani and Greenspan, 2002; Sarlani et al., 2004). Application of phasic rather than sustained noxious force utilsing von Frey Tests 2 and 3 duplicated such clinical findings, with both male and female rodents responding equivalently prior to neuropathic injury. Although both von Frey tests revealed the ability to detect heterogenous chronic pain with the level of mechanical allodynia found proportional to the number of sciatic sutures, as previously mentioned the adapted von Frey Test 3 was the only method to demonstrate exacerbated female mechanical hypersensitivity in recipients with significantly reduced pain (N1S3 recipients) or demonstrate that the rodent oestrus cycle influenced female mechanical allodynia.

Numerous avenues have been investigated to explain exaggerated female pain. One theorized explanation of the differences in pain response between the sexes is the hormonal milieu. The sex steroids estrogens, androgens and progesterones are primarily produced by the gonads from cholesterol, where their receptors have a wide distribution in the body including throughout the central nervous system (CNS) (McEwen and Alves, 1999; Aloisi, 2003; Craft, 2007). Oestrogen receptors specifically have been located in trigeminal neurons, dorsal root ganglion cells, as well as in brain areas such as the hypothalamus and the periaqueductal gray (PAG) (Shughrue et al., 1997; Yang et al., 1998; Kruijver et al., 2003; Merchenthaler et al., 2004; Bereiter et al., 2005; Loyd and Murphy, 2008) suggesting estrogen influences on both ascending and descending nociceptive pathways. This divergent localization of estrogen receptors enables a variety of functional roles within the CNS, which are not limited to the regulation of reproductive behavior. Despite modulating pain-processing systems such as the endogenous opioids (enkephalins) and GABA pathways (Amandusson et al., 1999; McEwen and Alves, 1999; Craft, 2007), the precise mechanisms underlying estrogen's role in pain remains unclear. Estrogens however, have also been demonstrated anti-inflamamtory, particular *in vitro* and are not the only sex hormones known to influence pain. Progesterone for example has also been demonstrated anti-inflamamtory in numerous diseases and in animal spinal cord injury models (Garcia-Ovejero et al., 2005; Muller and Kerschbaum, 2006; Labombarda et al., 2011). Although great inconsistency remains in the literature, there appears to be a strong association between the steroid hormones and pain.

Literature demonstrates prepubescent boys and girls display equal prevalence in the majority of chronic pain conditions, until puberty where a female prevalence is henceforward established until menopause (Ogura et al., 1985; Abu-Arefeh and Russell, 1994; Lipton et al., 2001; Sonmez et al., 2001; Bigal et al., 2007). Numerous studies have also demonstrated fluctuating hypersensitivity throughout the female menstrual (LeResche et al., 2003; Pamuk and Cakir, 2005; Crawford et al., 2009) and rodent oestrus cycles (Frye et al., 1992; Kayser et al., 1996; Giamberardino et al., 1997). Consequently, by assessing mechanical allodynia across a range of von Frey filaments using von Frey Test 3, this study's finding of an oestrus cycle effect replicates many other preclinical investigations as well as the clinical situation and indicates a hormonal sensitivity within the pain pathway.

Until recently, neuronal mechanisms were thought to solely contribute to pathological pain (Nicotra et al., 2012). Our understanding of chronic pain pathophysiology however, has since developed, with non-neuronal immune cells now also known to play role in both the initiation and maintenance of chronic pain (Haydon, 2001; Milligan and Watkins, 2009; Grace et al., 2011). Recent evidence demonstrates not only an interaction between steroid hormones and innate immune receptors known to play a role in chronic pain (Calippe et al., 2010; Loram et al., 2010), but exacerbated proinflammatory responses following steroid hormone-immune cell priming (Soucy et al., 2005; Rettew et al., 2009; Calippe et al., 2010). Considering the important role innate immune signaling plays in chronic pain processing, this interaction between neuroimmune function and the sex steroids may partially explain sex differences in pain sensitivity and requires further investigation (Nicotra et al., 2012).

The importance of utilizing preclinical techniques capable of reducing animal pain and stress has become increasingly apparent through the stringent statutory policies governing animal use in pain research (Gad, 1990). With chronic pain investigations in their very nature intentionally inflicting persistent pain, researchers face great difficulty in satisfying the Refinement category of the three R's unless ongoing refinement of experimental approaches continues. The ability of von Frey Test 3 to investigate both the role of sex and the influence of the rodent oestrus cycle utilizing the refined and novel graded model of neuropathy suggests that the use of the traditional CCI four-suture approach creates unnecessary suffering for the animal. In addition to reducing animal suffering, this refined preclinical methodology may also lead to the discovery and development of new analgesics. Existing pain models are limited in their binomial approach reducing the statistical power to investigate underlying pain mechanisms. Unlike currently utilized two group, sham controlled, and hence binary in nature pain models, which are more likely to reveal effective analgesics for the treatment of severe pain, the graded nerve injury model has the ability to detect heterogeneous pain, specifically moderate reductions in allodynia thereby unveiling potential analgesics for a range of chronic pain patients (Grace et al., 2010). Although utilizing several animals in order to generate the diverse pain treatment groups in this study, it is essential to note that this investigation extends the findings by Grace and Colleagues, demonstrating graded nerve injury in the female sex. In order to establish graded chronic pain for the first time in the female rodent, it was essential for this study to consider numerous pain treatment groups. The successful determination of sex and oestrus cycle effects without having to inflict maximal allodynia using von Frey Test 3, endorses the use of less allodynic animals in order to investigate chronic pain mechanisms in the future. Consequently therefore, although having to compromise the “Reduction” component of the three 3 R's in this particular investigation, using von Frey Test 3 has allowed “Refinement” in future investigations without disturbing the “Reduction” constituent. Consequently, in the hope of discovering novel analgesics and to preserve the use of animals in pain studies, this study commends the use of the graded model of graded sciatic nerve injury, specifically recommending the use of less allodynic animals in further preclinical pain research and the use of von Frey Test 3, which proved sensitive enough to identify contributing factors that other common von Frey tests could not reveal.

## Conclusion

To our knowledge, this study is the first to examine a role of sex and oestrus cycle in a refined heterogeneous allodynia model. In comparison to two commonly utilized von Frey testing methods, the adapted von Frey Test 3 whereby mechanical allodynia was assessed utilizing phasic application of lower threshold filaments, alone demonstrated a role for sex and the rodent oestrus cycle in female mechanical allodynia, without having to inflict maximal pain. Considering the emphasis animal ethics committee's place on the three R's and the difficulty pain researchers face in satisfying the “refinement” category to gain ethical approval, the ability to investigate chronic pain mechanisms using this modified von Frey approach in a model capable of inflicting less pain without compromising overall statistical significance is invaluable.

### Conflict of interest statement

The authors declare that the research was conducted in the absence of any commercial or financial relationships that could be construed as a potential conflict of interest.

## Appendix

**Table A1:** **Response rates for von Frey test 2 generated by the 2 g filament**.

	**PRO**	**OEST**	**MET**	**DI**
Pre-surgery	4.2 ± 0.30	5.0 ± 0.22	4.9 ± 0.24	4.7 ± 0.22
N0S0	5.2 ± 0.24	4.9 ± 0.18	4.1 ± 0.43	4.4 ± 0.44
N0S4	8.6 ± 0.44	6.5 ± 0.42	6.6 ± 0.56	9.7 ± 0.41
N1S3	20.0 ± 0.52	15.0 ± 0.46	14.3 ± 0.55	17.0 ± 0.51
N3S1	42.0 ± 0.44	41.0 ± 0.41	41.0 ± 0.38	43.0 ± 0.51
N4S0	54.0 ± 0.33	53.0 ± 0.44	51.0 ± 0.56	48.0 ± 0.53

*Responses generated utilizing von Frey Test 2 were examined following the traditional statistical approach whereby responses were independently examined at the 2 and 12 g von Frey filaments. For each predictor (sex, oestrus cycle phase, surgery and von Frey filament stimulus), the difference between the allodynia (percentage response for the 2 g von Frey filament) for each level of the predictor was estimated. This value obtained is referred to as the response rate, the percent response /30 (% response). The results presented throughout this study are expressed as the average difference in percent response, which are generated from the response rates*.

**Table A2:** **Response rates for von Frey test 2 generated by the 12 g filament**.

	**PRO**	**OEST**	**MET**	**DI**
Pre-surgery	8.3 ± 0.24	10.0 ± 0.22	9.6 ± 0.28	9.4 ± 0.21
N0S0	11.0 ± 0.21	10.0 ± 0.22	10.0 ± 0.26	11.0 ± 0.22
N0S4	22.0 ± 0.38	21.0 ± 0.28	22.0 ± 0.31	22.0 ± 0.26
N1S3	34.0 ± 0.32	32.0 ± 0.24	26.0 ± 0.38	34.0 ± 0.33
N3S1	61.0 ± 0.44	61.0 ± 0.51	64.0 ± 0.51	62.0 ± 0.57
N4S0	79.0 ± 0.51	79.0 ± 0.43	78.0 ± 0.44	81.0 ± 0.56

*Responses generated utilizing von Frey Test 2 were examined following the traditional statistical approach whereby responses were independently examined at the 2 and 12 g von Frey filaments. For each predictor (sex, oestrus cycle phase, surgery and von Frey filament stimulus), the difference between the allodynia (percentage response for the 2 g von Frey filament) for each level of the predictor was estimated. This value obtained is referred to as the response rate, the percent response /30 (% response). The results presented throughout this study are expressed as the average difference in percent response, which are generated from the response rates*.

**Table A3:** **Percent response values von Frey test 2**.

	**PRO**	**OEST**	**MET**	**DI**
Pre-surgery	6.9 ± 0.22	7.3 ± 0.18	7.3 ± 0.22	7.0 ± 0.16
N0S0	7.4 ± 0.20	7.0 ± 0.16	8.0 ± 0.22	8.7 ± 0.26
N0S4	17.0 ± 0.24	16.0 ± 0.11	17.0 ± 0.21	16.0 ± 0.26
N1S3	23.0 ± 0.33	25.0 ± 0.38	25.0 ± 0.41	22.0 ± 0.23
N3S1	48.0 ± 0.32	50.0 ± 0.48	51.0 ± 0.46	50.0 ± 0.41
N4S0	63.0 ± 0.44	64.0 ± 0.49	61.0 ± 0.33	41.0 ± 0.40

*Responses generated from von Frey Test 2 were used to examine the estimate of the slope of the linear relationship between the response and von Frey filament. The results presented throughout this study are expressed as the average difference in percent response, which are generated from the percent response values (the percent response /30 across von Frey filaments)*.

**Table A4:** **Response rates for von Frey test 3**.

	**PRO**	**OEST**	**MET**	**DI**
Pre-surgery	4.1 ± 0.20	4.9 ± 0.20	5.5 ± 0.80	5.1 ± 0.21
N0S0	5.1 ± 0.60	5.0 ± 0.72	5.2 ± 0.50	6.1 ± 0.60
N0S4	5.0 ± 0.44	6.1 ± 0.60	5.6 ± 0.30	5.3 ± 0.50
N1S3	9.8 ± 0.52	8.1 ± 0.52	7.7 ± 0.38	10.0 ± 0.60
N3S1	11.0 ± 0.42	8.9 ± 0.80	8.1 ± 0.80	11.0 ± 0.50
N4S0	12.0 ± 0.58	11.0 ± 0.74	11.0 ± 1.0	11.0 ± 0.85

*von Frey Test 3 examined the relationship between the response and the force of the von Frey filament. Results generated by von Frey Test 3 are expressed as the percent difference in response per increase in von Frey hair stiffness, which are generated from the estimated increase in percent response per increase in von Frey stiffness*.

## Abbreviations

CCI: chronic constriction injury
CNS: central nervous system
PAG: periaqueductal gray
PSNL: partial sciatic nerve ligation
N: sciatic nerve
S: subcutaneous
PO: postoperative
pro: pro-oestrus
oest: oestrus
met: metoestrus
di: dioestrus
AIC: Akaike's Information Criterion.
